# Modeling of recovery efficiency of sampling devices used in planetary protection bioburden estimation

**DOI:** 10.1128/aem.00832-23

**Published:** 2023-11-20

**Authors:** Michael DiNicola, Arman Seuylemezian, Lisa Guan, Christine Moissl-Eichinger, Amy Baker, Jason Johns

**Affiliations:** 1 Jet Propulsion Laboratory, California Institute of Technology, Pasadena, California, USA; 2 Medical University of Graz, Diagnostic and Research Institute of Hygiene, Microbiology, and Environmental Medicine, Graz, Austria; 3 SETI Institute, Mountain View, California, USA; 4 Herndon Solutions Group, Kennedy Space Center, Merritt Island, Florida, USA; Georgia Institute of Technology, Atlanta, Georgia, USA

**Keywords:** planetary protection, recovery efficiency, assay, bioburden

## Abstract

Planetary protection is a discipline dedicated to preventing biological contamination between celestial bodies and Earth during space exploration to ensure the integrity of scientific research. National space agencies such as the National Aeronautics and Space Administration and the European Space Agency set biological cleanliness requirements for their missions based on the guidelines provided in The Committee on Space Research’s Planetary Protection Policy. Compliance with these requirements is often demonstrated by surface sampling of spacecraft hardware and associated surfaces to enumerate the number of microorganisms present and establish that they are below the required level. The discipline has employed a variety of tools to perform sampling; the recovery efficiency of which is a key parameter used to generate spacecraft level cleanliness estimates. In this study, we investigated how recovery efficiency differs by inoculum amount and species. This was analyzed across different sampling devices using a set of microorganisms applied to stainless steel surfaces (representative of spacecraft surfaces) and two processing methods. Data were analyzed by developing a probabilistic model of the experimental process, from stainless steel coupon inoculation through recovery of spores observed in the form of colony forming units. The model quantifies the probability that an individual spore is recovered, a key metric for predicting bioburden and statistically assessing bioburden requirements. A cost function was developed to identify those assay methods that provided optimal bioburden estimation capability. Results show the nylon-flocked swab and the TX3211 wipe yield the highest recovery efficiency of those tested.

**IMPORTANCE:**

Planetary protection at the National Aeronautics and Space Administration (NASA) requires bioburden on certain spacecraft to be estimated via sampling in order to comply with biological cleanliness requirements. To achieve this, the recovery efficiency of devices used to sample the spacecraft pre-launch must be understood and their uncertainty quantified in order to produce the most reasonable estimates of bioburden. This study brings together experiments performed by NASA and the European Space Agency with approved swab and wipe sampling devices, inoculating steel coupons with laboratory strains of *Bacillus* spp. spores commonly recovered from spacecraft assembly clean rooms (*B. atrophaeus*, *B. megaterium*, *B. safensis* and *B. thuringiensis*), with a mathematical model of the assay process to assess recovery efficiency. The statistical treatment developed in this study allows comparison of bioburden estimates made from different devices processed by different methods. This study also gives stakeholders and practitioners a statistically rigorous approach to predict bioburden that can be folded into future modeling efforts.

## INTRODUCTION

The biotechnology and planetary protection group at the Jet Propulsion Laboratory and respective groups at the European Space Agency (ESA) have been monitoring the microbial spore bioburden of spacecraft and associated surfaces to minimize the inadvertent forward contamination of other planetary bodies and preserve the scientific integrity of exploratory missions. The practice is in compliance with the Outer Space Treaty (1), under a policy managed by the Committee on Space Research, and under agency level requirements imposed by the National Aeronautics and Space Administration (NASA) and the ESA. The primary devices used for acquiring samples of spacecraft hardware are sterile cotton swabs and polyester wipes. NASA uses the standard spore assay to intentionally select for cultivatable and hardy heat-shock tolerant bacterial spores on spacecraft hardware as a proxy for the total microbial bioburden (2, 3). The total spore count is used to assess whether spacecraft hardware has met spore bioburden standards set by Planetary Protection requirements.

Direct sampling takes place throughout the entire assembly life cycle of the mission through to launch (4). As such, a variety of surface materials are sampled and a diverse set of microorganisms are typically recovered (5). However, due to operational, logistical, and budgetary constraints, planetary protection engineers collect samples from a subset of the entire spacecraft surface. Surface counts are then extrapolated to estimate the total microbial bioburden and the bioburden density of the entire spacecraft in order to demonstrate compliance with launch requirements.

In order to accurately estimate the microbial bioburden present on the spacecraft surface, it is imperative to quantify the efficiency with which the sampling device recovers microorganisms. Previous studies aimed at quantifying the sampling device efficiency focused on a single device (6, 7) or several swab devices with only one test species (8). However, no studies to date have examined and compared the range of sampling devices currently in use in the planetary protection discipline. Moreover, these studies use different statistical techniques, none of which rigorously characterize the full uncertainty in the recovery process, and validation of the statistical analysis in these publications is not provided. In this comprehensive study, we examine all swabs and wipes currently approved by NASA and/or ESA for planetary protection use with a common modeling approach. In addition, we develop and validate a model of the entire end-to-end experimental process to capture uncertainties present from seeding of the stainless steel coupon surfaces, sampling of the surfaces, and wet laboratory processing. To understand the sensitivity of each sampling device to recovering different bacterial species, a range of species commonly recovered from spacecraft surfaces were used, primarily belonging to the commonly isolated *Bacillus* genera. NASA standard spore assay results from the Mars 2020 mission showed that 41% of isolated colony forming units (CFUs) from spacecraft surfaces identified as *Bacillus* at the genus level (9). The second most commonly isolated genera was *Priestia* which accounted for 16% of all isolated CFU. *Priestia* species formerly belonged to the *Bacillus* group but have now been reclassified as a separate genera. Non-spore forming organisms and non-culturable organisms are not addressed in this study as the focus is on the methodology used in planetary protection verification via the NASA or ESA spore assays.

Due to mitigation protocols that minimize microbial contaminants, typical spacecraft surface samples are extremely clean and samples that yield CFU are sparse. As such, these experiments focused on quantification of the device recovery efficiency with lower inoculum amounts to mimic the real-world application. Although a range of surface materials are sampled throughout the life cycle of each unique mission, stainless steel is a widely used material representative of a spacecraft surface; hence, it was the surface material of choice in this study and will have continued applicability for future missions.

**Fig 1:**
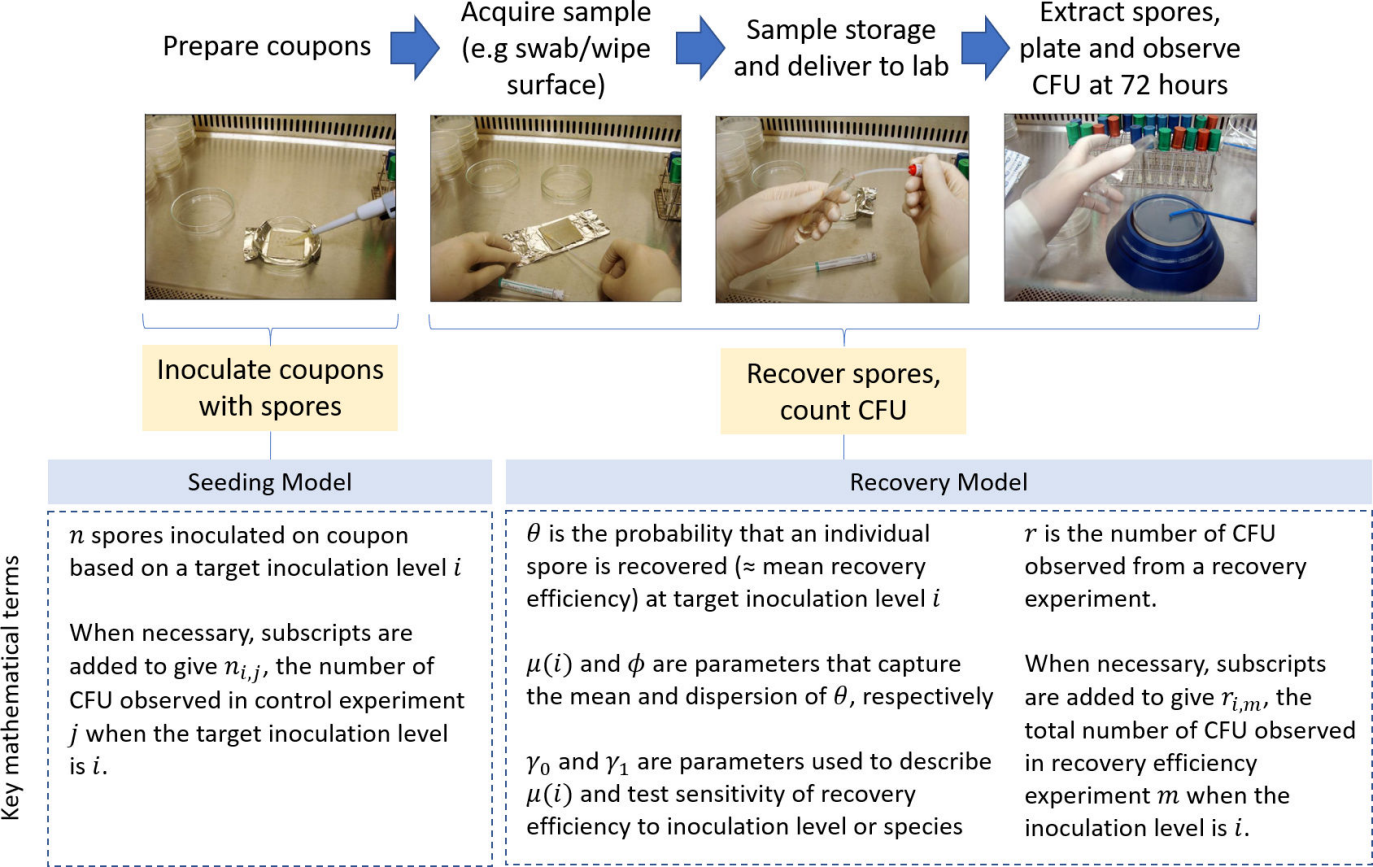
The seeding and recovery processes used in the swab recovery efficiency experiments of this study. (Left) A coupon is prepared by transferring a targeted number of spores from a stock solution of a known species onto the coupon (inoculation) and left to dry for 24 hours. Next, (middle) a sampling device (swab or wipe) is applied to the coupon, after which, it is contained and delivered to the lab for culture. Finally, (right) spores are extracted into a water solution which is then plated in Trypticase soy agar (TSA). Final CFU counts are made at 72 hours in culture, giving the observed data. This process is performed according to standard protocols (NASA or ESA standard assay). Key mathematical notation used in this study to model this process is shown at the bottom. A comprehensive list of all mathematical notation used in this study can be found in Appendix A.

Previous studies have demonstrated a statistical framework for performing bioburden accounting (10–12); however, they have not directly accounted for the recovery efficiency of the sampling devices used (13). The results communicated in this study will be integrated with the statistical framework currently under development (13) and will ultimately be used for performing bioburden accounting. Directly accounting for the recovery efficiency of these assay methods will provide a more accurate estimate of the total microbial bioburden and bioburden density originating from planetary protection sampling efforts. This statistical treatment also allows comparison of bioburden data estimates made from different devices and processed by different methods. Finally, we use the term “assay methods” throughout to refer to the process of acquiring a sample from a surface using a given sampling device, storing the sample and delivering it to the lab, extracting and plating spores, and observing CFU. The assay method for swabs is pictured in the spore recovery portion of Fig. 1, showing how coupons are inoculated with spores and the recovered spores are observed in the form of CFUs.

## MATERIALS AND METHODS

### Laboratory facilities

Experiments included in this study were conducted by two research groups in two distinct laboratories. All wipe experimental data were generated through NASA at Kennedy Space Center facilities. Swab data were generated through ESA at the Medical University Graz Center for Microbiome Research. Additionally, experiments using the Puritan cotton swab (the primary swab employed by NASA) were also conducted through NASA at the Kennedy Space Center facilities.

### Preparation of spores

A total of six different organisms were used in this study including *Bacillus (B.) atrophaeus* DSM 675, *B. safensis* DSM 19292, *B. megaterium* DSM 32, *B. megaterium* 2c1, *B. thuringiensis* DSM 2046, and *B. thuringiensis* E24. Spores from all six organisms were prepared following protocols highlighted in reference (14). Spore stocks were stored in 50% ethanol. *B. atrophaeus* was used as the representative organism for all of the experiments as it is the most commonly used *Bacillus* species of those listed for Planetary Protection studies (15). A sensitivity study was then performed with all six species to understand recovery efficiency differences across species.

### Sampling devices

Four different swab types were used in this study, including the Copan (Murrietta, CA) polyester (PE) ATP-free (170C) plain swab, Copan nylon-flocked swab (552C), Copan cotton swab (150CA), and the Puritan (Guilford, ME) cotton swab (806 WC). Both the Copan PE swab and the Copan cotton swab arrived gamma sterilized from the manufacturer. The Puritan cotton swab was sterilized in an autoclave and the Copan nylon-flocked swab was sterilized by exposure to ethlyene oxide.

For swab experiments, stainless steel coupons (No. 1.4301, 240 grain, Wilms Metallmarkt Lochnleche, Koln, Ehrenfeld, Germany) 1.5 × 50.5 × 50.5 mm in size were used. Stainless steel coupons were heat-sterilized for 3 hours at 160°C. Various concentrations of spores were spotted onto coupons in 4 µL droplets to minimize spread. Target inoculum levels are based on CFU observations from control experiments that quantified stock concentrations via culture plating. Coupons were then left to dry under laminar flow for 24 hours. A blank solution containing no spores was applied to negative control coupons which were also dried overnight. For consistent precision among recovery experiments conducted by both NASA and ESA, the number of replicates varied based on the initial inoculum amount and expected recovery, shown in Table 1. The experiments performed by ESA with the Copan cotton swab and by NASA with the Puritan swab did not include the case where the targeted inoculation level was 3 CFU per swab due to limits of detection in the experiment. All swab experiments testing the sensitivity of recovery efficiency to inoculum amount used the species *B. atrophaeus*. In both NASA and ESA swab experiments, sensitivity of recovery efficiency to species was addressed at the target inoculation level of 100 CFU per swab concentration.

Two different wipes were used in this study including the TexWipe (Kernersville, NC) TX3211 and TX3224 polyester wipes. Wipes were prepared for sampling by folding and rolling them into a 50 mL conical tube, and then adding 10 mL–20 mL of sterile water to saturate the wipe. The tube is then lightly capped and sterilized by autoclaving. The wipes are then allowed to cool to room temperature prior to sampling.

For wipe experiments, stainless steel metal coupons manufactured by the KSC Prototype Development Lab (Type 304 stainless steel; #4 brushed finish) and 16 inches × 16 inches × 0.04 inch in size were used. Coupons were autoclaved to ensure sterility. Various spore inoculum levels (Table 1) were then spotted onto coupons in 4 µL droplets. Target inoculum levels were quantified as they were for swab experiments. Given the low CFU counts typically observed from spacecraft surfaces, it can be assumed that spores are dispersed in monolayers. Therefore, all inoculum levels were well below the concentration needed to form multi-layered aggregates of spores (16). Coupons were left to dry overnight within a biosafety cabinet. A blank solution containing no spores was applied to negative control coupons which were also dried overnight. As with swab experiments, the number of replicates per wipe condition was varied based on the initial inoculum amount as shown in Table 1, and the sensitivity of wipe recovery efficiency to inoculum amount was tested using the species *B. atrophaeus*. To assess differences in recovery efficiencies of wipes by species, experiments were performed at a 400 spore inoculum per coupon concentration.

**TABLE 1:** Summary of sampling devices, processing techniques, and testing facilities used in this study^*a*^

Sampling device	Processing technique	Testing facility	Target inoculation level (CFU)	# of replicates
Copan PE swab	ESA standard	ESA	3, 5, 10, 15, 100	50, 40, 30, 20, 10
Nylon-flocked swab	ESA standard	ESA	3, 5, 10, 15, 100	50, 40, 30, 20, 10
Puritan cotton swab	NASA standard	ESA	3, 5, 10, 15, 100	50, 40, 30, 20, 12
Copan cotton swab	NASA standard	ESA	5, 10, 15, 100	40, 30, 20, 10
Nylon-flocked swab	NASA standard	ESA	3, 5, 10, 15, 100	20, 10, 10, 10, 10
Puritan cotton swab	NASA standard w/ Milliflex filter	NASA	5, 10, 15, 100	40, 30, 20, 10
TexWipe TX3211	NASA standard w/ Milliflex filter	NASA	16, 50, 160, 400	16, 8, 8, 8
TexWipe TX3224	NASA standard w/ Milliflex filter	NASA	16, 50, 160, 400	16, 8, 8, 8

^*a*^ For each of these cases, the target inoculation levels tested and respective number of replicates performed for each inoculation level are shown to the right. The experimental design reduces the number of replicates needed as the target inoculation level increases to maintain a consistent level of statistical accuracy across different inoculation levels throughout the experiment. Several technical replicates were still used per experimental parameter to understand error between replicates.

In addition to experimental conditions, several controls were used. Negative controls were performed by sampling sterile stainless steel coupons, positive controls were performed by direct plating of the spore solutions for each target inoculation level assessed.

#### Recovery of spores using swabs

Two different approaches were taken in processing the swabs, one followed the NASA standard protocol as outlined in NASA Handbook 6022 (17) and NASA-STD-8719.27 (2), the other followed the ESA standard protocol outlines in ECSS-Q-ST-70–55C (18).

Swab sampling procedures for both methods are similar, but there are slight differences in the sample processing protocol. For the ESA standard protocol, swabs are suspended in a phosphate-buffered saline (PBS) + Tween 80 solution, whereas for the NASA standard assay, swab heads are broken and suspended into sterile water. Both protocols then require vortexing and sonication, but the ESA assay typically spread plates 1 mL of the swab solution onto two separate R2A agar plates. In some instances, four plates with 0.5 mL solution in each plate were used to accommodate higher inoculation levels or when higher recovery efficiency was achieved. The NASA standard assay uses the pour plate method and deposits 2 mL of the swab solution into four Petri dishes and molten trypticase soy agar (TSA) (50°C) is poured on top. Plates from both assays were subsequently incubated at 32°C and colony counts were performed at 24, 48, and 72 hours.

Milliflex filtration was used for the Puritan cotton swab validation performed by the NASA facility. Plates were then left out to dry and subsequently incubated at 32°C. Colony were counted at 24, 48 and 72 hours.

#### Recovery of spores using wipes

Wipes were processed using only the NASA standard protocol as outlined in NASA Handbook 6022. After suspending the wipe in a rinse solution containing 85 mg/L potassium dihydrogen phosphate, 200 mg/L Tween 80; pH 7.2 and subjecting it to sonication and vortexing, the solution was then filtered through a 0.45 µm polyvinylidene difluoride (PVDF) membrane filter using the Millipore Milliflex membrane filtration system (Merck KGaAm Darmstadt, Germany) and plated onto Millipore filter cassettes (#RMHVMFX24) containing TSA. At first, approximately half of the wipe solution was poured through the filter. The remaining volume was then sonicated and vortexed for 5–10 seconds before pouring the solution through a second filter. The resulting plates were then incubated at 32°C and colony counts were performed at 24 and 48 hours. While the NASA standard assay protocol calls for final counts at 72 hours, a preliminary control experiment with wipes showed that no additional CFUs were observed passed 48 hours for any of the controls. Therefore, for the TX3211 wipes, CFUs were only observed for 48 hours.

### A mathematical model of recovery efficiency

The mathematical model to study recovery efficiency of various sampling devices and protocols considers the end-to-end process that generates the CFU data observed from each experiment, as described in Materials and Methods and as illustrated in Fig. 1. The experimental process is partitioned into two sub-models: an initial seeding model that captures the process of coupon preparation and inoculation; and a recovery model that captures the subsequent process of sampling, extraction and plating of spores, and counting of CFUs. Importantly, the recovery model is dependent on the outcome of the seeding model. Not capturing this dependency ignores the variability in the number of spores inoculated onto the coupon and has ramifications on how uncertainty is quantified for a given method’s recovery efficiency (see Appendix B.2). A bioburden estimation model and associated cost function are then developed to quantitatively compare methods as to their effectiveness in bioburden estimation applications. Stan software (19) in RStudio (20) was used to develop models and figures in software.

When developing the mathematical model, the notation p⁢(x∣y) is used to denote the conditional probability function of a random variable (or random vector) X taking the value x given another random variable (vector) Y takes the value y. When X is discrete, p⁢(x∣y) is understood to be a probability mass function; when X is continuous, p⁢(x∣y) is understood to be a probability density function. When evaluating this probability function at a specific value *x*_0_, we write $p\,\left(x=x_{0}|y\right)$. Finally, the unconditional probability function of a random variable (vector) X is denoted by p⁢(x). A comprehensive list of all other mathematical notation used in this study can be found in Appendix A.

#### Seeding model

The experimental process described in Materials and Methods begins with the preparation and inoculation of a coupon with a targeted number of spores, which we refer to as the target inoculation level (measured in CFU). As shown in reference (21), this process satisfies necessary independence and uniformity properties to be modeled by a Poisson distribution. In this study, we perform a rigorous statistical analysis of how the mean number of spores inoculated onto a coupon, λ, varies with the target inoculation level using observations from control experiments performed. (Control experiments performed for these recovery efficiency experiments inoculated growth medium directly.) In this study, it is assumed that exactly 1 CFU is generated by exactly one spore.

Let I be the set of target inoculation levels of interest and $J_{i}$ be the total number of control experiments performed at the target inoculation level i∈I. The probability that we observe $n_{i,j}$ CFU in the $j^{\text{th}}$ control experiment for the target inoculation level i given the parameter value λ is $p\,\left(n_{i,j}|\lambda \,\left(i\right)\right)=e^{-\lambda \,\left(i\right)}\,\frac{\lambda \,{\left(i\right)}^{n_{i,j}}}{n_{i,j}!}$, where λ follows a power law with respect to the target inoculation level; specifically, $\ln\left(\lambda \,\left(i\right)\right)=\delta_{0}+\delta_{1}\,\ln\left(i\right)$. Since the experimental design takes precautions to isolate coupons throughout the seeding process (e.g., biosafety hoods) and implements processes to avoid cross-contamination between experiments (e.g., usage of sterile pipettes between coupon inoculations), the number of CFU observed on coupons are treated as independent of one another by the model.

Therefore, the probability of observing all control experiment results $\text{n}=(n_{i,j}{)}_{j=1,i\in I}^{J_{i}}$ given the parameter values $\left(\delta_{0},\delta_{1}\right)$, is


(1) $$p(\text{n}|\delta_{0},\delta_{1})=\prod_{i\in I}\prod_{j=1}^{J_{i}}p(n_{i,j}|\lambda (i)).$$

The parameters $\left(\delta_{0},\delta_{1}\right)$ are given the joint prior distribution $p\,\left(\delta_{0},\delta_{1}\right)=p\,\left(\delta_{0}\right)\times p\,\left(\delta_{1}\right)$, where the prior distributions of $\delta_{0}$ and $\delta_{1}$ are independent normal distributions with means $\mu_{\delta ,0}$ and $\mu_{\delta ,1}$, and standard deviations $\sigma_{\delta ,0}$ and $\sigma_{\delta ,1}$, respectively. Independence is assumed in the prior distributions for simplicity and computational purposes, as there are enough data to uncover dependencies during fitting of the model. Since, prior to performing control experiments, the number of spores initially placed on a surface is expected to be centered around the target inoculation level, we set $\mu_{\delta ,0}=0$ and $\mu_{\delta ,1}=1$. We also set $\sigma_{\delta ,0}=5$ and $\sigma_{\delta ,1}=1$. Sensitivity analysis and prior predictive checks demonstrated that the model results were not significantly affected by a broad range of reasonable values for these parameters.

Using Bayes’ theorem to calculate the posterior distribution $p(\delta_{0},\delta_{1}|\text{n})$ from Equation (1), the posterior predictive distribution of the number of spores $\tilde{n}$ placed on a new coupon not in the control experiments, such as a coupon used to test recovery efficiency, when the target inoculation level is some positive integer $\hat{\iota }$, is given by


(2) $$p(\tilde{n}|\hat{\iota },\text{n})=\int_{\delta_{1}=-\infty }^{\infty }\int_{\delta_{0}=-\infty }^{\infty }p(\tilde{n}|\lambda (\hat{\iota }))\;p(\delta_{0},\delta_{1}|\text{n})d\delta_{0}\;d\delta_{1}\;.$$

Note that it may be the case that $\hat{\iota }\notin I$ and so Equation (2) can be used to predict the number of spores present on a coupon when other inoculation levels are targeted outside those used in the control experiments.

#### Recovery model

Suppose that a coupon has been inoculated with n spores of a specified species after targeting inoculation level i∈I, and that the probability of recovering an individual spore using a specific assay method is θ. It is assumed that inoculated spores will not die prior to sampling due to spore hardiness and their resistance to dessication (22). As in the seeding model, it is assumed in what follows that exactly 1 CFU is generated by exactly one spore. As with the seeding process, the design of the recovery experiments takes several measures to ensure uniformity of the sampled surface, sampling device, and assay protocol, and to avoid outside contamination: coupons are of the same material type and size and from the same manufacturer; reagents are tested for sterility according to manufacturer recommendations; sampling devices are applied to surfaces; and spores are extracted from these devices, plated in growth medium and cultivated, and CFU counted following prescribed protocols (NASA or ESA standard assays). These measures help ensure that all spores have the same probability of recovery, θ, for a given assay method. Moreover, sonication and vortexing of the spore stock prior to inoculation of coupons and during spore extraction minimize such phenomena as microorganism clumping, which allow observations of CFU to be treated independently of one another in the modeling. With these assumptions in place, the probability that r CFU are observed from a total of n spores on a coupon is given by


(3) p(r∣n,θ)=(nr)θr(1−θ)n−r,

which is recognized as the binomial distribution. This distribution has the property that the expected value of the number of recovered spores $E\,\left[R\right]=\sum_{r=0}^{n}r\,p\,\left(r|n,\theta \right)=n\times \theta$, or equivalently, $\theta =\frac{E\,\left[R\right]}{n}=E\,\left[\frac{R}{n}\right]$, which is the mean recovery efficiency. Experimental observation of the number recovered allow us to estimate E⁢[R] by a sample mean $\bar{r}$ which, by the law of large numbers, will converge to E⁢[R] as the sample size increases, implying that $\theta \approx \frac{\bar{r}}{n}$. Hence, when n is known, the probability that an individual spore is recovered can be approximated by the mean recovery efficiency calculated from experiment.

In order to consider potential over-dispersion in this study’s recovery data, we will allow θ to be realizations from a beta distribution parameterized by a mean value μ and dispersion parameter *ϕ*


(4) $$p(\theta |\mu (i),\phi )=\frac{\Gamma (\phi )}{\Gamma (\mu (i)\phi )\Gamma ((1-\mu (i))\phi )}\theta^{\mu (i)\phi -1}(1-\theta {)}^{(1-\mu (i))\phi -1}\;,$$

where Γ is the gamma function. The beta distribution provides a robust statistical approach to capture uncertainty in spore recovery owing to deviation from certain “ideal” conditions where most of the variation is due to the experimental design (e.g., number of replicates). These ideal conditions are often not realistic when performing microbiological experiments, where there are significant uncontrollable aspects of the experimental subjects (e.g., diversity within spore species) and how they interact with the materials used in the experiment (e.g., how spores adhere to surfaces), which can add significant variation beyond what a simpler model can accommodate. Using the terminology of probability theory, the spores may not be identically distributed, leading to dispersion not captured by these simpler models. The parameter ϕ of the beta distribution in Equation (4) captures this additional dispersion.

In this study, we would also like to assess whether or not a trend exists between the mean recovery efficiency and the target inoculation level. To do this, we further let be a function of $\gamma_{0}$ and $\gamma_{1}$, using the logit transformation, $\mu (i)=\frac{1}{1+e^{-(\gamma_{0}+i\gamma_{1})}}$, which has found broad applicability in bio-assay research (23). Hence, given that a coupon is originally inoculated with n spores after targeting an inoculation level of *i*, the probability that *r* CFU are observed using a specified assay method is ∫θ=01(nr)θr(1−θ)n−rp(θ∣μ(i),ϕ)dθ, which simplifies to give


(5) p(r∣n,μ(i),ϕ)=(nr)Γ(ϕ)Γ(μ(i)ϕ)Γ((1−μ(i))ϕ)Γ(r+μϕ)Γ(n−r+(1−μ(i))ϕ)Γ(n+ϕ),

which is recognized as the beta-binomial distribution. Due to the beta distribution used to model θ, the beta-binomial distribution models a process that would otherwise be adequately described by a binomial distribution, but where additional variation is caused by factors out of the control of the experimental design. As discussed in the previous paragraph, these factors are typically related to variability in biologically related features of microorganisms or how they interact with materials. The added variation is inversely related to the parameter ϕ, with the process converging to a binomial distribution as ϕ goes to infinity.

The number of spores inoculated onto a coupon does not depend on the recovery method or protocol as is made explicit by its lack of dependence on the parameters μ⁢(i) and ϕ. Moreover, Equation (2) gives us a model of the number of spores inoculated onto a coupon used for the recovery experiments when the target inoculation level is i. Hence, we have that p⁢(n∣μ⁢(i),ϕ)=p⁢(n) and define $p(n)\equiv p(\tilde{n}=n|\hat{\iota },\text{n})$. Therefore, the probability that r CFU are observed using a specified sampling device and protocol given parameter values μ⁢(i) and ϕ is


(6) $$p(r|\mu (i),\phi )=\sum_{n=r}^{\infty }p(n)\;p(r|n,\mu (i),\phi ).$$

Turning to the actual experiments performed using a given assay method, for each inoculation level targeted i∈I and each of $m=1,\ldots ,M_{i}$ independent experiments performed at that target inoculation level, there were a total of $r_{i,m}$ CFU observed during the recovery process. The probability of observing $\text{r}=(r_{i,m}{)}_{m=1,i\in I}^{M_{i}}$ CFU across all independently performed experiments is


(7) $$p(\text{r}|\gamma_{0},\gamma_{1},\phi )=\prod_{i\in I}\prod_{m=1}^{M_{i}}p(r_{i,m}|\mu (i),\phi )\;.$$

Recall that μ⁢(i) is a function of the parameters $\gamma_{0}$ and $\gamma_{1}$. The parameters $\left(\gamma_{0},\gamma_{1},\phi \right)$ are given the joint prior distribution $p\,\left(\gamma_{0},\gamma_{1},\phi \right)=p\,\left(\gamma_{0}\right)\times p\,\left(\gamma_{1}\right)\times p\,\left(\phi \right)$. Here, $\gamma_{0}$ and $\gamma_{1}$ are given normal distributions and ϕ is given an exponential distribution with rate parameter κ (note that dispersion is strictly positive). For the same reasons discussed in the Seeding model section, independence is assumed in the joint prior distribution even though dependence is known to exist among regression coefficients. When designing the experiments, the mean recovery efficiency was assumed to be 50% to first order, with a broad degree of uncertainty. Given this information prior to doing the experiments, we set the means for γ parameters $\mu_{\gamma ,0}=\mu_{\gamma ,1}=0$ so that the mean of μ⁢(i) is $\frac{1}{2}$ for all i, and set $\sigma_{\gamma ,0}=5$ and $\sigma_{\gamma ,1}=1$ to allow for considerable variability as to the value of μ⁢(i) and the trends it allows with the target inoculation level. The value of κ was set to $\frac{1}{1,000}$ to allow the data to more strongly inform the dispersion parameter, ϕ. Sensitivity analysis demonstrated that the model results were not significantly affected by a broad range of reasonable values for these parameters.

Using Bayes’ theorem to calculate the posterior distribution $p(\gamma_{0},\gamma_{1},\phi |\text{r})$ from Equation (7), the posterior distribution of θ when the target inoculation level is $\hat{\iota }$ is calculated by integrating over all possible values of $\mu (\hat{\iota })$ and ϕ:


(8) $$p(\theta |\hat{\iota },\text{r})=\int_{\gamma_{1}=-\infty }^{\infty }\int_{\gamma_{0}=-\infty }^{\infty }\int_{\phi =0}^{\infty }p(\theta |\mu (\hat{\iota }),\phi )\;p(\gamma_{0},\gamma_{1},\phi |r)d\gamma_{0}d\gamma_{1}d\phi .$$

Note that we have used the fact that $\gamma_{0}$, $\gamma_{1}$ , and ϕ do not depend on $\hat{\iota }$ and that, once $\mu \,\left(\hat{\iota }\right)$ and ϕ are known, no further information is provided by the data r when determining the distribution of θ. Finally, the 95% credibility interval from the marginal posterior distribution of $\gamma_{1}$ was used to test for significance of a trend between recovery efficiency and target inoculation level. If this interval contained 0, then it was concluded that there is insufficient evidence to include a trend parameter in the model at this time. Otherwise, it was concluded that the dependency between recovery efficiency and inoculation level should be further investigated.

#### Bioburden estimation

Since sampling devices and assay protocols have uncertain efficiencies that do not account for 100% of spores on surfaces, bioburden needs to be estimated using probabilistic methods. Estimating bioburden utilizes much of the theory developed in the Seeding model and Recovery model sections. A sampling device, such as a swab or wipe, and assay method, such as the NASA standard assay, are applied to sample a surface. The sampling device is then contained in a sterile container and transported to a lab for extraction, plating, and culture. Within a specified time range, CFUs are counted and recorded. Key differences between this process and the one described in the previous sections are:

1. In real applications, there can be many different species of microorganisms present on surfaces, with differing recovery characteristics.
2. The number of microorganisms on the surface prior to sampling is not experimentally controlled, but is dependent on the facility’s air flow properties, human activity in the facility, and other phenomenon, some of which are poorly understood.

To consider (1), we introduce the notion of a microorganism “recovery type.” Two individual microorganisms are of the same recovery type if they share the same probability of being recovered. Let $\pi_{k}$, for k=1,…,K, represent the probability that an individual microorganism is of recovery type k, and let $\theta_{k}$ be the probability that an individual microorganism is recovered given it is of type k.

Once microorganism species are identified, two species might be considered to be of the same recovery type if the difference between their probabilities of being recovered is statistically insignificant as judged by the 95% credibility interval. Since current assay protocols do not identify microorganism species upon culture, the probability that CFUs are observed from Equation (3) conditioned on knowing the number of microorganisms present on the surface and the probabilities of an individual microorganism being of a given recovery type becomes


(9) p(r∣n,π1,…,πK,θ1,…,θK)=(nr)(∑k=1Kπkθk)r(1−∑k=1Kπkθk)n−r,

where, for all k, $\pi_{k}\in \left[0,1\right]$ and $\sum_{k=1}^{K}\pi_{k}=1$ , and Equation (9) equals zero when r>n. In this formulation, the last recovery group K is reserved for the set of “novel” microorganisms that have unknown recovery efficiency probability relative to the sampling device or protocol being applied, and $\pi_{K}=1-\sum_{k=1}^{K-1}\pi_{k}$. The recovery probability for an individual microorganism of a given type k is $p(\theta_{k}|n,{\text{r}}_{k})$ as calculated by Equation (8), where ${\text{r}}_{k}$ is the vector of experimental observations of the recovery efficiency of recovery type k microorganisms relative to a given assay method. When k=K, it will be assumed that $\theta_{k}$ follows the distribution in Equation (4) with $\mu =\frac{1}{2}$ and ϕ=1, also referred to as a Jeffreys prior distribution. (In this study, we assume that $\pi_{k}$ are known, fixed quantities for all k. This model readily accommodates the situation where these parameters are unknown or need to be estimated themselves from prior knowledge or data.)

Informing the parameters $\pi_{k}$ and $\theta_{k}$ for general applications is outside the scope of this study. Additional experiments and modeling would need to be performed to analyze the relative abundance of different microorganisms in the sampling facility in order to inform $\pi_{k}$. The same experimental design and modeling approach used in this study can be used to determine the recovery efficiency, $\theta_{k}$, of each type of microorganism once identified. For purposes of this study, it is assumed that $\pi_{1}=\frac{2}{3}$ of microorganisms have a recovery type similar to *B. atrophaeus* and $\pi_{1}=\frac{1}{3}$ of the microorganisms are of a novel recovery type (24).

To consider (2), this study assumes that fallout of microorganisms onto surfaces follows a Poisson distribution with mean parameter, λ. We assume ignorance of the correct Poisson model, and so we let λ follow a Jeffreys prior distribution proportional to $\lambda^{-\frac{1}{2}}$, although a more physics-based model would better inform this probability. Under these assumptions, the probability that there are n microorganisms on the surface is $p_{0}(n)\propto \frac{\Gamma (n+\frac{1}{2})}{\Gamma (n+1)}$ prior to the observation of recovery data. (In this case, the Jeffreys prior is improper, which leads to an improper prior on n. However, because a Poisson likelihood multiplied by this Jeffreys prior can be bounded by a gamma distribution, it can be shown that this leads to a proper probability density for all results discussed in this study.)

Continuing to treat individual microorganism recoveries as independent, the probability that r CFUs are observed from Equation (3), when there are n microorganisms on the surface and the probability of an individual microorganism being of a given type is known, is


(10) $$\begin{array}{c} p\left(r|n,\pi_{1},\ldots ,\pi_{K}\right)\\ =\int_{\theta_{1}=0}^{1}\cdots \int_{\theta_{K}=0}^{1}p\left(r|n,\pi_{1},\ldots ,\pi_{K},\theta_{1},\ldots ,\theta_{K}\right)\prod_{k=1}^{K}p\left(\theta_{k}|n,r_{k}\right)d\theta_{1}\cdots d\theta_{K}. \end{array}$$

By Bayes’ theorem, the probability that there are n microorganisms on the surface when r CFU are observed is


(11) $$p(n|r)\equiv p(n|r,\pi_{1},\ldots ,\pi_{K})\propto p(r|n,\pi_{1},\ldots ,\pi_{K})\times p_{0}(n)\;.$$

Note that this formulation allows estimation of bioburden when the CFU observed is zero without the experimenter having to introduce unnecessary (and maybe problematic) conservatism into their bioburden results by rounding them to an arbitrary positive value such as 1, as has been common practice (25). Moreover, if the distribution of r for this assay method is known to be p⁢(r) for surfaces of similar size, composition, and microbial population, the unconditional probability of n can be calculated and the probability that there are n microorganisms on a surface is $p\,\left(n\right)=\sum_{r=0}^{\infty }p\,\left(n|r\right)\,p\,\left(r\right)$. This can be used when a sampling event has not or cannot be performed on the surface.

The model given by Equation (11) is used in the next section to compare different assay methods in the common scenario where 0 CFU are observed by the recovery process, i.e., r=0. This model can also be used to predict bioburden when sampling hardware surfaces: provided a number of CFU observed by way of a sampling event together with the relative abundance of each species and corresponding recovery efficiency, a probability distribution of the number of spores on the surface at the time of sampling is output by Equation (11). While this latter application is not the immediate focus of this study, it is of great utility to any engineering or public health discipline performing assays, particularly those facing low biodensity environments.

#### Comparing methods for purposes of bioburden estimation

In what follows, we will index each assay method by an integer q=1,…,Q, and refer to assay method q as ${ℳ}_{q}$. We will also index the probability functions above by q to make clear what method is being evaluated. For instance, the probability functions in Equation (11) will be written $p_{q}\,\left(n|r\right)$ to make clear that these probabilities (and all calculations and data going into these probabilities) are relative to method q.

Equation (11) allows us to develop an objective metric to compare the sampling devices and protocols of this study in the context of bioburden estimation. This metric is the expected value of a “cost function.” This cost function evaluates the recovery efficiency of a given assay method relative to some reference method, ${ℳ}_{0}$. Ideally, we would have a method ${ℳ}^{*}$ that has a mean probability of 100% that an individual microorganism is recovered, with zero variability—a method that recovers everything from a surface and is perfectly reliable in doing so—and set ${ℳ}_{0}={ℳ}^{*}$. In particular, method ${ℳ}^{*}$ implies that the number of microorganisms on a surface sampled, n, equals the number of CFU observed from the sample, r, with probability of 1.0. We measure the cost of deviating from this ideal using the square difference between the modeled bioburden n from method ${ℳ}_{q}$ and the bioburden estimated from the ideal method ${ℳ}^{*}$, $C\,\left({ℳ}_{q},n|{ℳ}^{*},r\right)={\left(n-r\right)}^{2}$. To compare different methods, we take the expected cost over all possible bioburden estimates, n, when the observed CFU is equal to r, that is, $E_{r}^{*}\,\left(q\right)\equiv E\,\left[C\,\left({ℳ}_{q},n|M^{*},r\right)\right]=\sum_{n=r}^{\infty }{\left(n-r\right)}^{2}\,p_{q}\,\left(n|r\right)$, which can be simplified to reveal how it trades the mean probability that an individual microorganism is recovered with its variability:


(12) $$E_{r}^{*}(q)=\sigma_{q,r}^{2}+{\left(\mu_{q,r}-r\right)}^{2}\;,$$

where $\mu_{q,r}=\sum_{n=r}^{\infty }n\,p_{q}\,\left(n|r\right)$ is the expected number of spores on a surface given r have been recovered from method q and $\sigma_{q,r}^{2}=\sum_{n=r}^{\infty }{\left(n-\mu_{q,r}\right)}^{2}\,p_{q}\,\left(n|r\right)$ is the variance of the number of spores on a surface given r have been recovered from method q. In Equation (12), $\sigma_{q,r}^{2}$ is directly influenced by the variance in the method’s recovery efficiency, penalizing methods with higher variability in their probability of individual CFU recovery. The term ${\left(\mu_{q,r}-r\right)}^{2}$ is a direct consequence of the difference between the mean probability of individual spore recovery of method q with that of the ideal, ${ℳ}^{*}$. This term essentially penalizes a method with a smaller mean recovery efficiency. The cost function tells us how to trade these two—variability and mean recovery efficiency—when comparing the recovery efficiency of different methods.

A particular case of interest we will use in this study is when r=0. This will give us the metric $E_{0}^{*}$ to compare assay methods for what is by far the most common case in spacecraft bioburden monitoring, where 0 CFU are observed in the recovery process. Hence, this metric conveys preference for method q over $q^{\prime }$ when $E_{0}^{*}(q)<E_{0}^{*}(q^{\prime })$.

## RESULTS AND DISCUSSION

The mathematical model of the experimental process described in Materials and Methods is applied to analyze recovery efficiency. Recovery efficiency was modeled and analyzed holistically with the assay technique deployed; therefore, assessments of recovery efficiency are bound to both the sampling device utilized as well as the assay methodology used. Our results for recovery efficiency are described in terms of θ, the probability that an individual microorganism is recovered, since this is the relevant parameter for bioburden estimation. This term is roughly equivalent to the mean recovery efficiency for the reason given in the Recovery model section, and this more familiar phrasing will be used when discussing results. In this section, we first present results having to do with the sensitivity of recovery efficiency to inoculation level and species, and then summarize overall recovery efficiency results. Finally, we will discuss validation of the mathematical model developed in this study as well as issues of over-dispersion in the data. The modeling presented in this study addresses shortcomings of previous recovery efficiency studies, which tend to apply confidence intervals or other standard statistical metrics to characterize uncertainty in recovery efficiency rather than a model of the end-to-end assay process. Notably, validation is completely missing from other studies, a key test of the realism of any statistical analysis.

### Sensitivity of recovery efficiency to inoculation level and species

**Fig 2:**
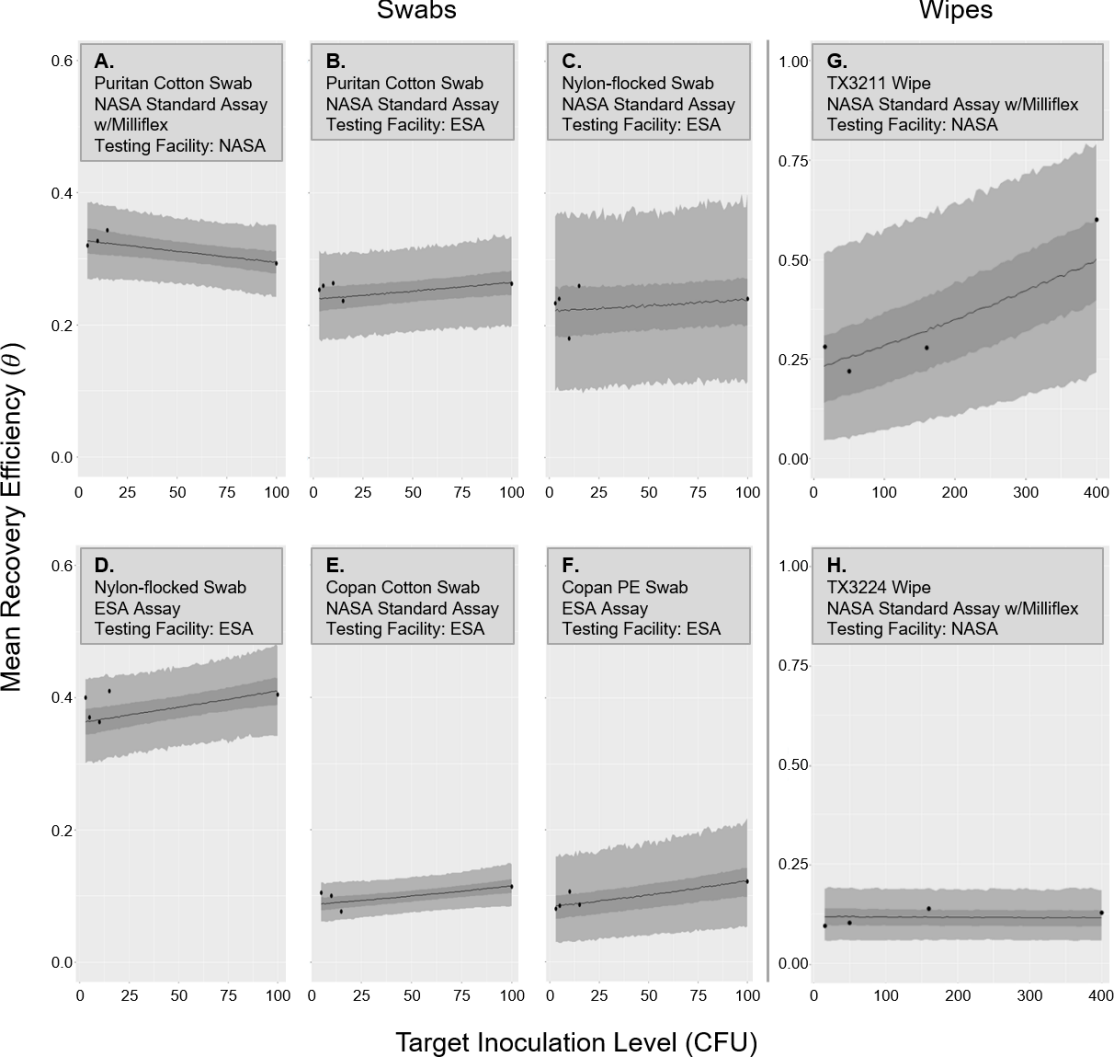
The mean recovery efficiency (probability of an individual spore being recovered, θ) with respect to the target inoculation level for swab experiments (**A–F**) and wipe experiments (**G, H**) performed in this study with *B. atrophaeus*. The mean value of θ is shown by the solid line; 50% and 95% credibility intervals for θ are shown by darker and lighter gray ribbons, respectively (ragged edges of ribbons are due to simulation variation). Calculations of the mean recovery efficiency from experiment are shown by black dots.

We address the sensitivity of recovery efficiency to inoculation level by testing if there is a statistically significant trend in the recovery efficiency of *B. atrophaeus* with respect to the target inoculation level. As shown in Fig. 2, panels A–F, recovery efficiency does not have a strong dependence on inoculation level for swabs. No trend is statistically significant as judged by the 95% credibility interval of the parameter $\gamma_{1}$, although borderline cases exist in panels B and F of the figure. Note that experiments underlying panels A and E did not test at the target inoculation level of 3 CFU due to detection limit concerns. A lower number of replicates were used in the experiments underlying panel C as compared to other experiments, leading to larger scatter in the results relative to other cases.

Panels G and H of Fig. 2 show similar graphics for the two wipes assessed by this study. When observations with a target inoculation level of 400 CFU are included in the model, there is an evident trend that is statistically significant as judged by the 95% credibility interval of the parameter $\gamma_{1}$ for the TX3211 wipe (Fig. 2G). Recovery efficiency at the target inoculation level of 400 CFU are of less practical value to bioburden estimation applications (e.g., Mars-bound missions) since this result many times leads to cleaning of the hardware and resampling. In fact, removing these observations from the data results in no trend being statistically significant. However, there are cases where these higher inoculation levels may be important (e.g., Europa Clipper), and target inoculation levels up to 1,000 CFU should be considered in the future. The trend observed in (Fig. 2G) indicates that the TX3211 wipe’s recovery efficiency may increase at some target inoculation level between 160 and 400 CFU. In contrast, the TX3224 wipe (Fig. 2H) appears to provide a much more stable, albeit lower recovery efficiency than the TX3211 wipe.

**Fig 3:**
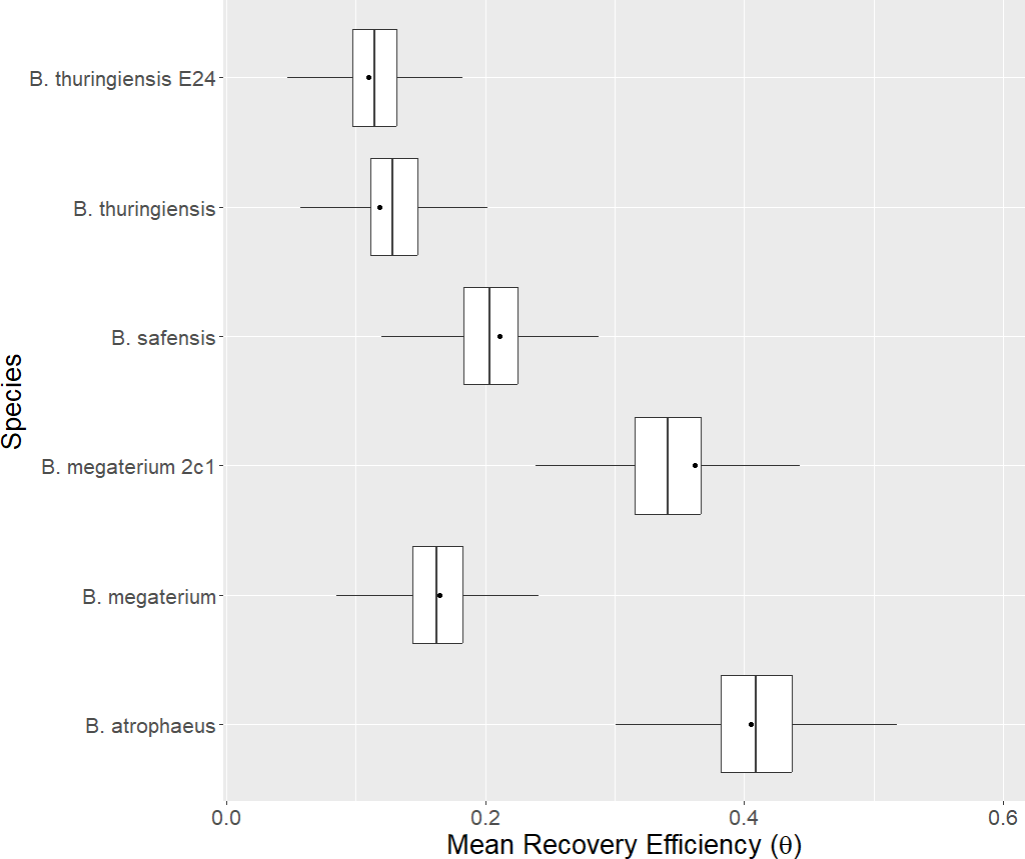
The mean recovery efficiency (probability of an individual spore being recovered, θ) with respect to species for the nylon-flocked swab; target inoculation level = 100 CFU. Box and whisker plots show the modeled middle 50% and 95% credibility intervals, respectively. The model’s mean value is shown by the solid vertical line in the middle of the box. Mean recovery efficiencies calculated from experiment are shown by black dots.

There are significant differences in the recovery efficiency when the sampling device is applied to different species. Fig. 3 shows results for the nylon-flocked swab. The reasons for variations in recovery efficiencies are unclear, but it seems possible that different physicochemical adhesive properties, like hydrophobicity or the biomolecular composition of spore sheaths, can affect the release of spores from surfaces (26). Other swabs and wipes show similar behavior in recovery efficiency with respect to species, although it is difficult to discern differences in the TX3224 recovery efficiency with respect to species. See Appendix B.1 for further discussion.

### Recovery efficiency summary

The mean recovery efficiency of *B. atrophaeus* and expected cost of each assay method studied are summarized in Table 2 for swabs and Table 3 for wipes. We analyze these groups separately because they are applied in different contexts, with a wipe being applied to much larger surfaces than a swab. The values in these tables were calculated assuming that no trend exists between recovery efficiency and inoculation level, as was demonstrated above in the Results and Discussion. Also, while the expected cost presented here captures several important quantitative characteristics of recovery efficiency, it does not consider other factors that are also relevant to making decisions as to which sampling device or protocol to use. For instance, the practicality of using a particular device and assay methodology, handling constraints, and accessibility controls in place to ensure the safety of hardware are not considered by this metric (e.g., electrostatic discharge and particle shedding properties). Resources required to revise processes or change from one standard protocol to another are also not considered.

**TABLE 2:** Mean recovery efficiency and expected cost for swab sampling devices assessed in this study using *B. atrophaeus^a^*

Identifier	Sampling device	Processing technique	Testing facility	Mean recovery efficiency	Expected cost
A	Puritan cotton swab	NASA standard w/ Milliflex filter	NASA	31% (26%, 36%)	7
B	Puritan cotton swab	NASA standard	ESA	25% (19%, 31%)	10
C	Nylon-flocked swab	NASA standard	ESA	23% (12%, 36%)	17
D	Nylon-flocked swab	ESA standard	ESA	38% (32%, 45%)	4
E	Copan cotton swab	NASA standard	ESA	10% (8%, 13%)	60
F	Copan PE swab	ESA standard	ESA	10% (3%, 18%)	76

^*a*^ Values in parentheses are the endpoints of a 95% credibility interval.

**TABLE 3:** Mean recovery efficiency and expected cost for wipe sampling devices assessed in this study^*a*^

Identifier	Sampling device	Processing technique	Testing facility	Mean recovery efficiency	Expected cost
G	TX3211 wipe	NASA standard w/ Milliflex filter	NASA	27% (6%, 56%)	27
H	TX3224 wipe	NASA standard w/ Milliflex filter	NASA	12% (9%, 16%)	46

^*a*^ Values in parentheses are the endpoints of a 95% credibility interval.

For swabs, the nylon-flocked sampling device using the ESA processing technique and facility has the lowest expected cost, and is therefore the swab method preferred by the modeling. This method also has the highest mean recovery efficiency with moderate variability, driving it to have a lower expected cost relative to other methods. A noticeable drop in the mean recovery efficiency occurs with ESA facility nylon-flocked swab experiments when using the NASA (C) instead of ESA (D) standard assay. This result is of borderline statistical significance at the 5% level. Fewer replicates performed for (C) lead to higher variability in the mean recovery efficiency results. Making the number of replicates consistent with (D) would allow this difference to be better quantified. The Puritan cotton swab was the second most preferred sampling device. There is a borderline statistically significant difference (at the 5% level) between its mean recovery efficiency when tested at a NASA (A) versus ESA (B) facility. However, it is unclear whether this is due to testing facility or processing technique, as the NASA facility used Milliflex filtration when plating which was not done at ESA. Further experiments controlling for processing technique and testing facility would be needed in order to better understand the mechanism causing this difference. Finally, both Copan swabs (E and F) performed worst in these experiments, with mean recovery efficiencies significantly lower and expected costs much higher than other swab assay methods.

Of the two wipes assessed in this study, the TX3211 wipe has the lowest cost and is therefore the wipe method preferred by the modeling. Despite having much higher variability in its mean recovery efficiency than the TX3224 wipe, the TX3211 wipe has ∼2 times the recovery efficiency on average, driving down its expected cost in this comparison.

### Model validation and dispersion

**Fig 4:**
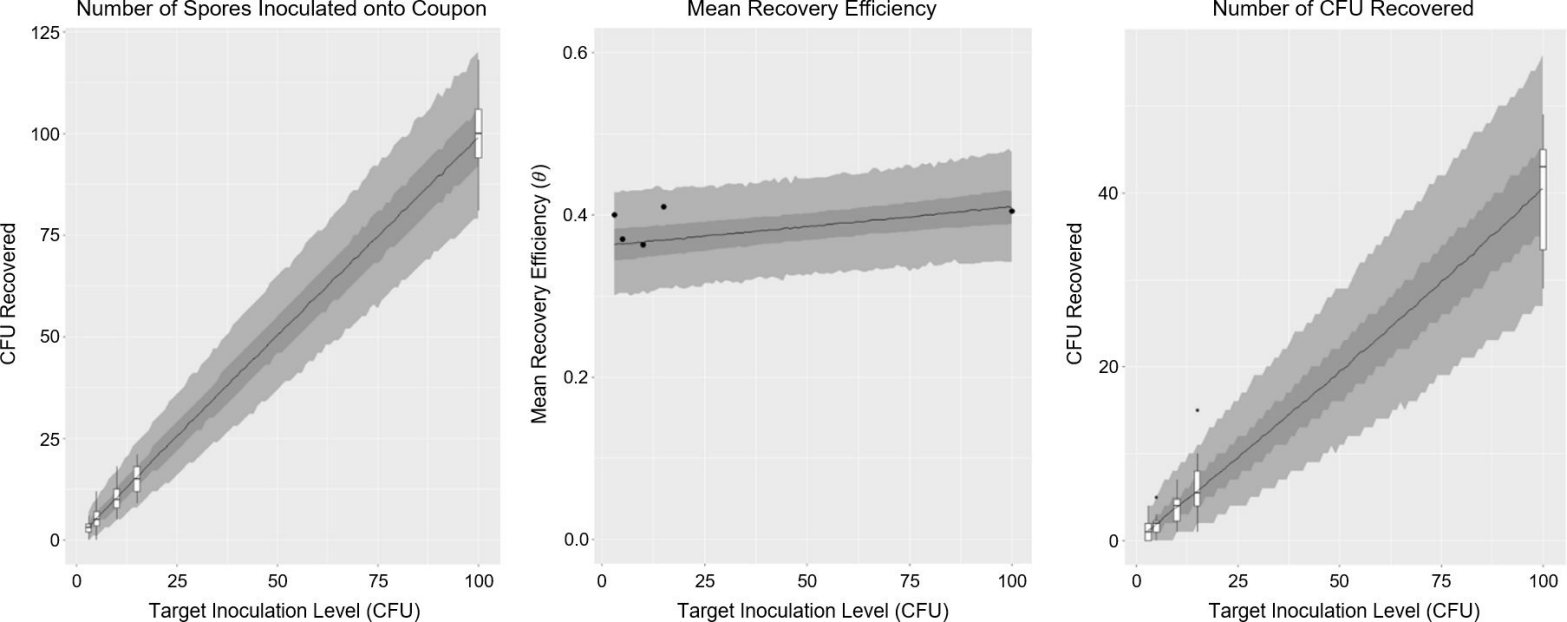
Validation of the mathematical model developed in this study for the nylon-flocked swab (*B. atrophaeus*, ESA facility, ESA standard method). On the left, the seeding model predictions (gray ribbons and solid line) are compared with the actual data observations (box-whisker plots) from controls. The center graphic shows the mean recovery efficiency (probability of individual spore recovery, θ) with model predictions (gray ribbons and solid line) compared with recovery efficiencies calculated from experiment (black dots). On the right, the integrated seeding and recovery model predictions (gray ribbons and solid line) are compared with the actual data observations (box-whisker plots) from recovery efficiency experiments. The mean value of the model is shown by the solid line; 50% and 95% credibility intervals of the model are shown by darker and lighter gray ribbons, respectively (ragged edges of ribbons are due to simulation variation). Box and whisker plots show the middle 50% and 95% ranges, respectively, calculated from the data. The model validates very well with observation, capturing the dispersion in the observed data.

The seeding and recovery models developed in Materials and Methods were validated by assessing how well they predict the observations from the actual experiments performed. Fig. 4 shows very good agreement between model and observation. Further validation to assess the prediction accuracy of the model using data not part of this study’s model calibration is ongoing.

**Fig 5:**
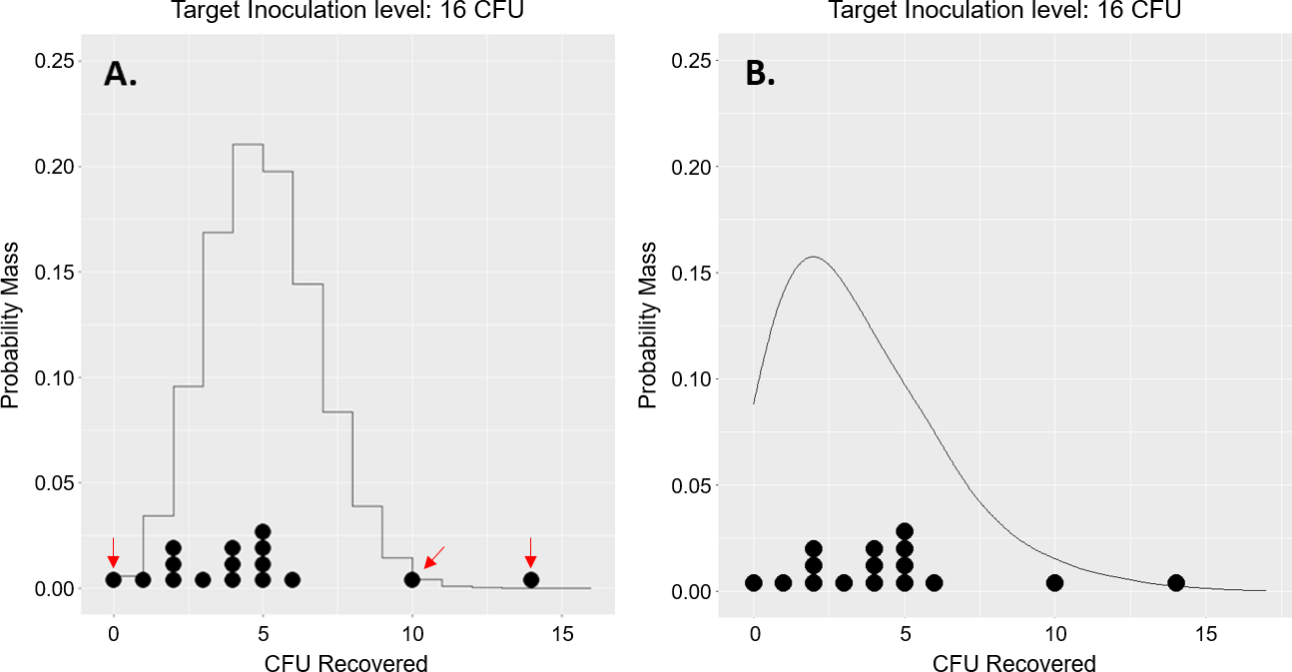
Over-dispersion in a simpler model (**A**) warranting a more complex model (**B**) to capture this additional variability in the data. Red arrows in (**A**) at 0, 10, and 14 observed CFU point to clear evidence of over-dispersion. The curves in each plot show model predictions, which is overlaid with a dot plot of the actual observations made from experiment (TX3211 wipe, target inoculation level = 16 CFU).

A primary reason why the model validates well is due to how it captures dispersion in the observations from experiment. In this case, the added model complexity of including the parameter ϕ is warranted due to several outlier observations that occur in simpler models, leading to over-dispersion. Fig. 5 demonstrates this in the case of the TX3211 wipe, but is representative of other assay methods assessed by this study. The model in case A, a simpler binomial model assigns almost zero probability to the observations at 0, 10, and 14 CFU (pointed out by red arrows), whereas the model in B (the model used in this study) does a better job of capturing this variability. Similar results were observed for most other swabs and wipes at other inoculation levels targeted. Note that the model developed in this study captures this dispersion statistically, but does not explain the phenomenon. Further experimentation is required to uncover the mechanisms causing this dispersion. Reasons for this dispersion are hypothesized to come from non-uniformities in experiment, such as surface roughness of surfaces sampled.

Finally, treating the number of spores inoculated onto a coupon as a fixed value (e.g., equal to the average of positive controls, which is common practice) can also lead to misrepresenting the variability in the mean recovery efficiency and inducing bias. This is discussed further in Appendix B.2. Several previous studies (6–8), utilizing common statistical metrics (e.g., standard deviation), binomial statistics, or fixing the number inoculated onto the coupon when constructing confidence intervals, may be vulnerable to these over-dispersion and bias issues discussed in this section.

### Conclusion

In this study, we have assessed the recovery efficiency of various sampling procedures using a combination of sampling devices and assay processing techniques. A mathematical model was built and informed by controlled experiments (i) to determine a ranking between sampling devices and recovery techniques and (ii) to provide a framework for integration of recovery efficiency to existing Bayesian statistical pipelines used to calculate bioburden density. The nylon-flocked swab and TX3211 wipe perform best for purposes of bioburden estimation. For most practical purposes, there does not appear to be a strong trend between recovery efficiency and the inoculation level, although there appears to be an ∼2× increase in recovery efficiency of the TX3211 wipe once exceeding a certain target inoculation level between 160 and 400 CFU. However, given the stringent bioburden requirements imposed on current planetary protection sensitive missions (<300 CFU/m^2^ for most spacecraft surfaces), a vast majority (∼80%) of planetary protection CFU counts per sample are either 0 or 1 CFU. Further characterization of this increase in recovery efficiency for the TX3211 wipe may be of value to potential future missions with less stringent requirements.

This study shows sensitivity of recovery efficiency to *Bacillus* spores across all sampling devices certified for use with spacecraft surfaces, but further experimentation and better knowledge of the distribution of species in clean rooms are necessary to integrate this variability into bioburden estimation models. The development of molecular-based microbial detection techniques may play a critical role in providing a more comprehensive distribution of species present. Additional controlled studies comparing sampling devices (particularly wipes) using both NASA and ESA protocols as well as Milliflex filtration vs standard plating processes may strengthen existing data sets and help improve the mathematical modeling. Finally, the mathematical modeling developed in this study provides the foundation for a rigorous probabilistic tool that can be made available to microbiologists when assessing recovery efficiency and performing bioburden estimation of low biomass analytical samples. This model can be adapted for a range of applications where low culture counts must be used as a representative sample for a broader assessment of cleanliness, such as within the healthcare or biodefense fields. With this tool, bioburden estimates made from different devices or processed by different methods can be compared, and assay protocols and sampling devices can be ranked in order of their bioburden estimation capability.

## Figures and Tables

**Figure A1:**
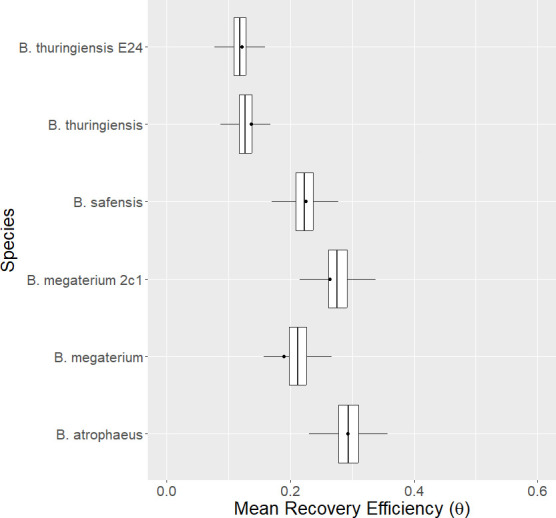
The mean recovery efficiency (probability that an individual spore is recovered, θ) with respect to species for the Puritan cotton swab using the NASA Standard processing technique; target inoculation level = 100 CFU. Box and whisker plots show the modeled middle 50% and 95% credibility intervals, respectively. The model’s mean value is shown by the solid vertical line in the middle of the box. Mean recovery efficiencies calculated from experiment are shown by black dots.

**Figure A2:**
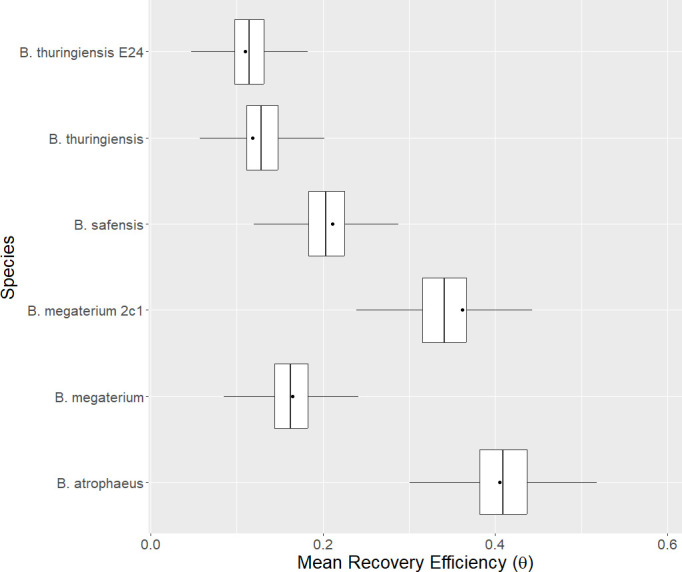
The mean recovery efficiency (probability that an individual spore is recovered, θ) with respect to species for the nylon-flocked swab using the ESA Standard processing technique; target inoculation level = 100 CFU. Boxbox and whisker plots show the modeled middle 50% and 95% credibility intervals, respectively. The model’s mean value is shown by the solid vertical line in the middle of the box. Mean recovery efficiencies calculated from experiment are shown by black dots.

**Figure A3:**
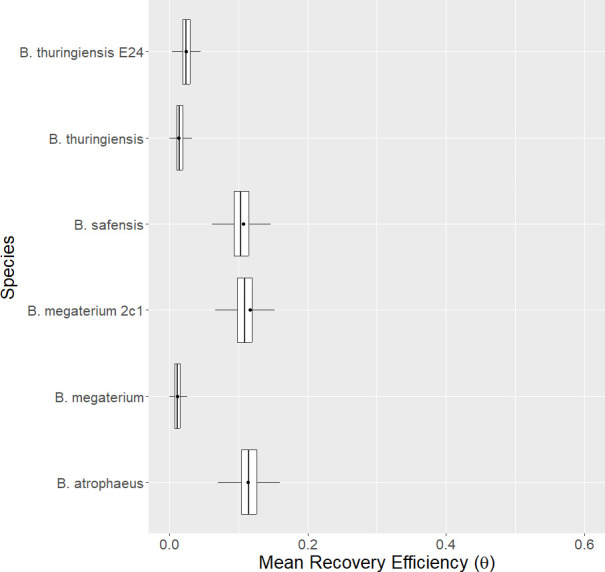
The mean recovery efficiency (probability that an individual spore is recovered, θ) with respect to species for the Copan cotton swab using the NASA Standard processing technique; target inoculation level = 100 CFU. Boxbox and whisker plots show the modeled middle 50% and 95% credibility intervals, respectively. The model’s mean value is shown by the solid vertical line in the middle of the box. Mean recovery efficiencies calculated from experiment are shown by black dots.

**Figure A4:**
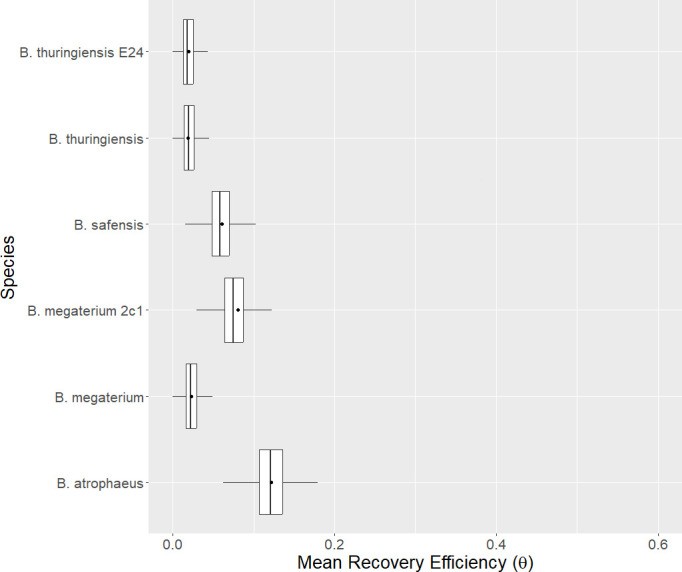
The mean recovery efficiency (probability that an individual spore is recovered, θ) with respect to species for the Copan PE swab using the ESA Standard processing technique; target inoculation level = 100 CFU. Box and whisker plots show the modeled middle 50% and 95% credibility intervals, respectively. The model’s mean value is shown by the solid vertical line in the middle of the box. Mean recovery efficiencies calculated from experiment are shown by black dots.

**Figure A5:**
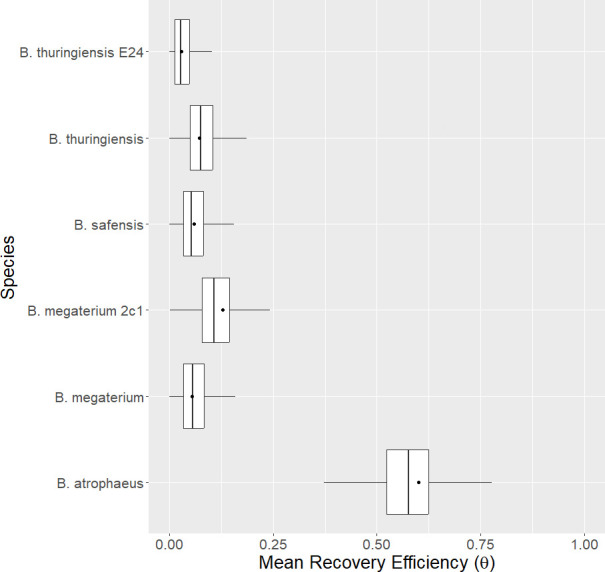
The mean recovery efficiency (probability that an individual spore is recovered, θ) with respect to species for the TX3211 wipe using the NASA Standard processing technique with Milliflex filtration; target inoculation level = 400 CFU. Boxbox and whisker plots show the modeled middle 50% and 95% credibility intervals, respectively. The model’s mean value is shown by the solid vertical line in the middle of the box. Mean recovery efficiencies calculated from experiment are shown by black dots.

**Figure A6:**
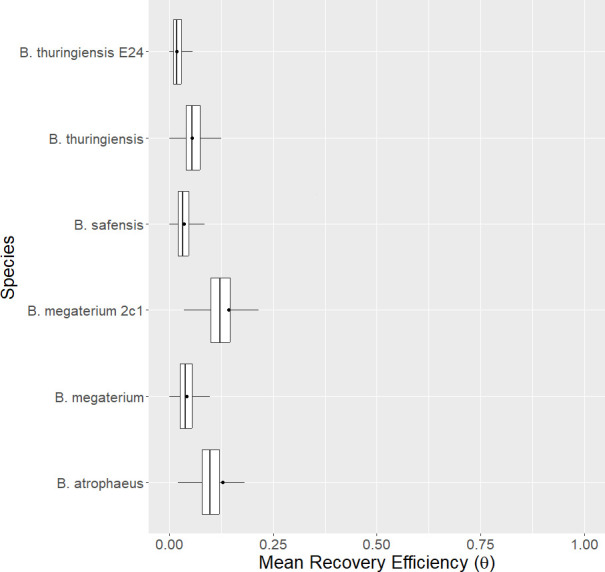
The mean recovery efficiency (probability that an individual spore is recovered, θ) with respect to species for the TX3224 wipe using the NASA Standard processing technique with Milliflex filtration; target inoculation level = 400 CFU. Boxbox and whisker plots show the modeled middle 50% and 95% credibility intervals, respectively. The model’s mean value is shown by the solid vertical line in the middle of the box. Mean recovery efficiencies calculated from experiment are shown by black dots.

**Figure A7:**
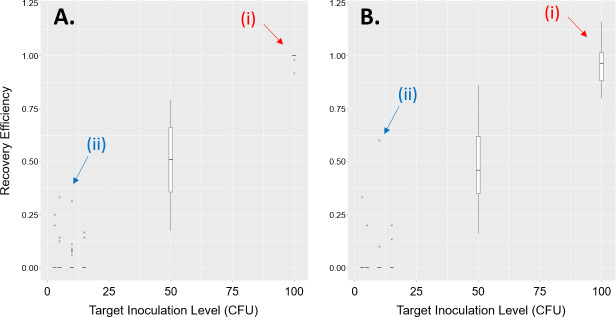
Simulated recovery efficiency experiments with a strong increasing trend with inoculation level. Panel (A) shows box plots of the actual recovery efficiency ratios. Panel (B) shows box plots of the same ratios but with a denominator fixed at a single value (the mean number of CFU observed from control experiments at each target inoculation level). The box represents the inter-quartile range, the whiskers the 95% data range, and outliers denoted by “×” symbols. The mean value is indicated by the horizontal line in the box. Arrows for (i) and (ii) draw attention to the bias and general disruption of the variance structure in Panel (B) relative to Panel (A).

**Figure A8:**
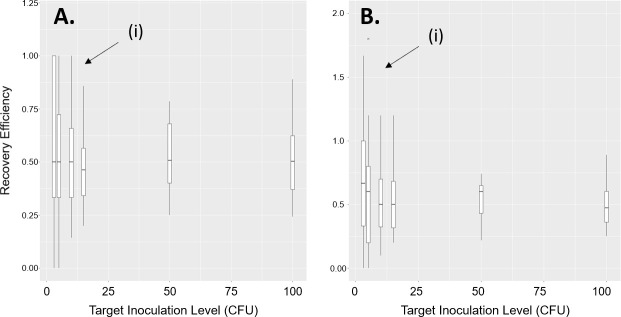
Simulated recovery efficiency experiments with no trend with inoculation level and an average recovery efficiency of 50%. Panel (A) shows box plots of the actual recovery efficiency ratios. Panel (B) shows box plots of the same ratios but with a denominator fixed at a single value (the mean number of spores inoculated onto the coupon at each target inoculation level). The box represents the inter-quartile range, the whiskers the 95% data range, and outliers denoted by “×” symbols. The mean value is indicated by the horizontal line in the box. Arrows for (i) draw attention to the significant bias and disruption of the variance structure in Panel (B) relative to Panel (A) at lower inoculation levels.

## SUPPLEMENTAL MATERIAL

Stan code aem.00832-23-s0001.txt Stan code for seeding and recovery models.

README aem.00832-23-s0002.txt Outline of Supplemental Material to "Modeling of Recovery Efficiency of Sampling Devices used in Planetary Protection Bioburden Estimation."

Supplemental Data Table S1: Recovery Efficiency Experiments aem.00832-23-s0003.csv A database containing all information recorded from recovery efficiency experiments used for the recovery model.

Supplemental Data Table S2: Positive Control Experiments aem.00832-23-s0004.csv A database containing all information recorded from positive controls performed for the seeding model.

ASM does not own the copyrights to Supplemental Material that may be linked to, or accessed through, an article. The authors have granted ASM a non-exclusive, world-wide license to publish the Supplemental Material files. Please contact the corresponding author directly for reuse.

## Appendices

These appendices include a summary of the mathematical notation used in this study (Appendix A), further discussion of the mathematical model, including additional discussion associated with the sensitivity of recovery efficiency to different species (Appendix B.1) and a brief discussion of how uncertainty in the inoculation level can affect statistical calculations of recovery efficiency (Appendix B.2), and discussion of Stan code and R packages used to implement the model developed for this study (Appendix C).

## Appendix A: Mathematical Notation

The notation for the mathematical model is summarized below, first for the seeding model, then for the recovery model, and finally for bioburden estimation and method comparison models.

### Seeding model notation

 I: the set of target inoculation levels of interest; i∈I denotes a particular target inoculation level.

 $J_{i}$: the total number of control experiments performed for the seeding process at the target inoculation level, i. Each control experiment is indexed by $j=1,\ldots ,J_{i}$.

$n_{i,j}$: the number of CFU observed in control experiment j when the target inoculation level is i.

**n**: the vector of all observations $n_{i,j}$ from control experiments for the number of spores inoculated onto a coupon.

λ(i): the mean number of spores inoculated onto a coupon as a function of the target inoculation level, i.

$\delta_{0}$: intercept parameter for power law behavior assumed for λ⁢(i).

$\mu_{\delta ,0}$: mean of a normal prior distribution on the parameter $\delta_{0}$.

$\sigma_{\delta ,0}$: standard deviation of a normal prior distribution on the parameter $\delta_{0}$.

$\delta_{1}$ slope parameter for power law behavior assumed for λ⁢(i).

$\mu_{\delta ,1}$: mean of a normal prior distribution on the parameter $\delta_{1}$.

$\sigma_{\delta ,1}$: standard deviation of a normal prior distribution on the parameter $\delta_{1}$.

$\hat{\iota }$: the target inoculation level, potentially not in I.

$\tilde{n}$: the predicted number of spores inoculated onto a new coupon not in control experiments (such as those used to test recovery efficiency).

### Recovery model notation

 $M_{i}$: the number of recovery efficiency experiments performed at target inoculation level, i; each recovery efficiency experiment is indexed by $m=1,\ldots ,M_{i}$.

θ: the probability that an individual spore is recovered at target inoculation level i using a specific assay method.

n: the number of spores inoculated onto a coupon prior to applying a specific assay method.

r: the number of CFU observed from a recovery experiment.

$\bar{r}$: the sample mean of the number of CFU observed from recovery efficiency experiments for a specific assay method.

Γ(⋅): the Gamma function; that is, if x is a positive real number, $\Gamma (x)\equiv \int_{0}^{\infty }u^{x-1}e^{-u}\;du$.

μ(i): the mean value of the probability that an individual spore is recovered as a function of the target inoculation level, i.

ϕ: the dispersion parameter associated with the probability that an individual spore is recovered.

$\gamma_{0}$: the intercept parameter used in a logit transformation describing how the probability that an individual spore is recovered is related to the target inoculation level.

$\gamma_{1}$: the slope parameter used in a logit transformation describing how the probability that an individual spore is recovered is related to the target inoculation level.

$r_{i,m}$: the total number of CFU observed in recovery efficiency experiment m when the target inoculation level is i.

**r**: the vector of all observations $r_{i,m}$ from recovery efficiency experiments.

$\mu_{\gamma ,0}$: mean of a normal prior distribution on the parameter $\gamma_{0}$.

$\sigma_{\gamma ,0}$: standard deviation of a normal prior distribution on the parameter $\gamma_{0}$.

$\mu_{\gamma ,1}$: mean of a normal prior distribution on the parameter $\gamma_{1}$.

$\sigma_{\gamma ,1}$: standard deviation of a normal prior distribution on the parameter $\gamma_{1}$.

κ: the rate parameter for an exponential prior distribution on parameter ϕ.

### Bioburden estimation model notation

 K: the number of microorganism recovery types; each type is indexed by k=1,…,K.

$\pi_{k}$: the probability that an individual microorganism is of type k.

$\theta_{k}$: the probability that an individual microorganism of type k is recovered.

n: the number of microorganisms on a surface that will be assayed.

λ: the mean number of microorganisms on a surface that will be assayed.

### Comparing methods for purposes of bioburden estimation notation

 Q: the number of distinct assay methods being compared; each type is indexed by q=1,…,Q.

$M_{q}$: assay method q.

$M_{0}$: a reference assay method, identified by the subscript 0.

$M^{*}$: the ideal assay method with perfect recovery efficiency; 100% probability of recovering a microorganism with zero variability.

$C(M_{q},n|M_{q^{\prime }},r)$: the cost of deviating from method $q^{\prime }$ when r CFU are observed using method q resulting in a bioburden estimate of n spores.

$E_{r}^{*}(q)$: the expected cost of method q as compared to the ideal method ${ℳ}^{*}$ when r CFU are observed during the recovery process.

$\mu_{q,r}$: the expected number of spores on a surface given r CFU have been observed from method q.

$\sigma_{q,r}^{2}$: the variance of the number of spores on a surface given r CFU have been observed from method q.

## Appendix B: Additional Discussion of the Mathematical Model

### Sensitivity of recovery efficiency to species

The following charts show the sensitivity of recovery efficiency to different species, by sampling device: the Puritan cotton swab using the NASA Standard processing technique (Fig. A1), the nylon-flocked swab using the ESA Standard processing technique (Fig. A2), the Copan cotton swab using the NASA Standard processing technique (Fig. A3), the Copan Polyester (PE) swab using the ESA Standard processing technique (Fig. A4), the TX3211 wipe using the NASA Standard processing technique with Milliflex filtration (Fig. A5), and the TX3224 wipe using the NASA Standard processing technique with Milliflex filtration (Fig. A6). The target inoculation level for each experiment was 100 CFU. The species tested were *B. atrophaeus*, *B. megaterium*, *B. megaterium 2*c1, *B. safensis*, *B. thuringiensis* and *B. thuringiensis E24*.

In general, *B. atrophaeus* has the highest recovery efficiency. In many cases, the *B. megaterium 2*c1 recovery efficiency was very close to that of *B. atrophaeus*. The only exception to this is the TX3224 wipe, where there is not as much sensitivity of recovery efficiency to species. For swabs, *B. megaterium* has a significantly lower recovery efficiency than *B. megaterium 2c1. B. safensis* has a recovery efficiency closer to *B. megaterium 2*c1 in all cases except for the nylon-flocked swab, in which case the recovery efficiency was closer to *B. megaterium*. Differences in recovery efficiency between *B. thuringiensis* and *B. thuringiensis E24* are not statistically significant (at the 5% level). The sensitivity of recovery efficiency of wipes is not as apparent for wipes. In the case of the TX3211 wipe, *B. atrophaeus* has significantly higher recovery efficiency than other species tested. As stated in the main publication, the reasons for these differences in recovery efficiency are not well understood and require further study.

### Brief discussion of how the uncertainty in the spores inoculated onto a coupon can affect statistical calculations

The seeding model devloped in Materials and Methods captures uncertainty in the coupon seeding process prior to application of the sampling device. Common practice is to use control experiments counting CFU to estimate the mean number of spores inoculated onto a coupon, and then use the mean of the CFU observed in these controls as the denominator when calculating recovery efficiency percentages. Not only does this allow for nonsensical recovery efficiency values above 100%, but it also ignores the variability and bias this induces into estimates of recovery efficiency. In this section, we use a computer program of the model developed in the main paper to simulate observed CFU from recovery efficiency experiments. We then apply the common practice approach to analyzing the recovery efficiency of these simulated experiments to illustrate the issues mentioned above.

Panel A of Fig. A7 shows a box-whisker plot of a computer simulated set of recovery efficiencies. Here, we have simulated a set of recovery efficiency experiments for a hypothetical sampling device at target inoculation levels of 3, 5, 10, 15, 50 and 100, according to the model described in the main publication. The box represents the inter-quartile range, the whiskers the 95% data range, and outliers denoted by “×” symbols. The mean value is indicated by the horizontal line in the box. Replicates at each target inoculation level for recovery experiments are similar to what were done in the real experiments of this study. The number of spores inoculated onto a coupon at each target inoculation level follows a negative binomial distribution as was the case in the real swab experiments of this study. A strong increase in efficiency with inoculation level is imposed by the model on these simulated experiments to draw attention to certain behaviors (this is accomplished by setting the model parameters $\gamma_{0}=-5.0,\gamma_{1}=0.1$, and ϕ=10.0). For instance, the behavior pointed to in (i) of Panel A shows how recovery efficiencies approaching 100% are highly skewed to high recovery efficiencies, while (ii) shows how very low inoculation levels have highly skewed recovery efficiencies towards zero. Note the large number of outliers at these lower inoculation levels, a result of calculating ratios with small integers. That is, the lower the inoculation level the larger the change in recovery efficiency for each observed CFU. Note that this simulation allows for zero spores to be inoculated onto the coupon (more likely for low inoculation levels); in this case the recovery efficiency ratio is undefined as is not included on the plot, which induces some bias to statistics derived from this data.

Panel B shows the resulting recovery efficiencies recorded from the hypothetical experiment if the number inoculated onto these hypothetical coupons is treated as a fixed value at the mean of (simulated) controls. As can be seen in (i) of Panel B, many observations show recovery efficiencies greater than 100%, which is nonsensical (contamination is not possible in these simulated experiments). Moreover, the variance structure is greatly disturbed as much of the skewness from Panel A (i) is lost. Similarly, (ii) of Panel B points to outliers at low inoculation levels, the variance structure becoming more discretized relative to Panel A when the denominator is held fixed. Therefore, any statistics derived from data that fixes the number of spores inoculated onto the coupon (as in Panel B) will contain bias and could grossly misrepresent the variance structure of the actual data.

Fig. A8 shows similar data from another set of simulated experiments, this time with no sensitivity to inoculation level and a mean recovery efficiency of 50% ($\gamma_{0}=0,\gamma_{1}=0,\phi =10.0$). Panel B shows significant bias at lower inoculation levels and drastic deformation of the variance structure when fixing the number of spores inoculated onto the coupons to the mean of control experiments. Nonsensical recovery efficiency ratios above 1.0 occur in this panel about 25% of the time for the inoculation level of three CFU. These biases and distortions of variance become even more evident as the mean recovery efficiency is increased ($\gamma_{0}>0$).

Both panels in each figure bias our statistical analysis and muddle the quantification of uncertainty for recovery efficiency (panels A less so that panels B). This can be avoided by not basing our statistics on recovery efficiency ratios, but instead modeling them according to the process by which the data were generated in the first place; that is, the seeding and recovery processes modeled in the main body of this study.

## Appendix C: Stan Code and R Packages used for the Modeling in this Study

The model was developed in R (version 4.1.0) in the RStudio environment (version 1.4.1717), using Stan (version 2.19.2).

The attached .txt file contains the Stan code for the seeding and recovery models. Note that, when the seeding model is integrated with the recovery model in Equation (6), a negative binomial distribution is used to approximate p⁢(n), the posterior predictive distribution of the number of spores inoculated onto a coupon given by Equation (2). Also, a finite upper bound, equal to the 99.9th percentile of the negative binomial representation of the seeding model posterior distribution, was used as the upper limit of the sum in Equation (6). These approximations were made for computational reasons; sensitivity analysis showed that the results were not significantly affected.

Stan code was run in the RStudio environment using the rstan (version 2.21.3) package and the function stan to run the Hamiltonian Monte Carlo to find the posterior distribution of model parameters. Default number of chains (chains = 4), iterations per chain [iter = 2,000, warmup = floor(iter/2)] and default controls (e.g., adapt_delta = 0.8) were used when running the seeding model. All chains appropriately mixed and results sufficiently converged with these default settings (e.g., no divergent transitions, Rhats = 1.00).

For the recovery model, all default settings were used when running Stan, with the exception that the step size used in the Hamiltonian Monte Carlo needed to be increased to avoid divergent transitions (adapt_delta = 0.99). With these settings, all chains appropriately mixed and results sufficiently converged.

Data for each model came from the data sets included in the supplementary materials to this study. Graphics and other calculations performed in R used the packages tidyverse (version 1.3.1) and dplyr (version 2.1.1).
